# Rat whisker motor cortex is subdivided into sensory-input and motor-output areas

**DOI:** 10.3389/fncir.2013.00004

**Published:** 2013-01-28

**Authors:** Jared B. Smith, Kevin D. Alloway

**Affiliations:** ^1^Department of Neural and Behavioral Sciences, Penn State UniversityHershey, PA, USA; ^2^Center for Neural Engineering, Penn State UniversityUniversity Park, PA, USA

**Keywords:** motor cortex, neuronal tracing, sensorimotor, intracortical microstimulation, somatosensory cortex

## Abstract

Rodent whisking is an exploratory behavior that can be modified by sensory feedback. Consistent with this, many whisker-sensitive cortical regions project to agranular motor [motor cortex (MI)] cortex, but the relative topography of these afferent projections has not been established. Intracortical microstimulation (ICMS) evokes whisker movements that are used to map the functional organization of MI, but no study has compared the whisker-related inputs to MI with the ICMS sites that evoke whisker movements. To elucidate this relationship, anterograde tracers were placed in posterior parietal cortex (PPC) and in the primary somatosensory (SI) and secondary somatosensory (SII) cortical areas so that their labeled projections to MI could be analyzed with respect to ICMS sites that evoke whisker movements. Projections from SI and SII terminate in a narrow zone that marks the transition between the medial agranular (AGm) and lateral agranular (AGl) cortical areas, but PPC projects more medially and terminates in AGm proper. Paired recordings of MI neurons indicate that the region between AGm and AGl is highly responsive to whisker deflections, but neurons in AGm display negligible responses to whisker stimulation. By contrast, AGm microstimulation is more effective in evoking whisker movements than microstimulation of the transitional region between AGm and AGl. The AGm region was also found to contain a larger concentration of corticotectal neurons, which could convey whisker-related information to the facial nucleus. These results indicate that rat whisker MI is comprised of at least two functionally distinct subregions: a sensory processing zone in the transitional region between AGm and AGl, and a motor-output region located more medially in AGm proper.

## Introduction

The functional organization of rodent motor cortex (MI) has traditionally been defined by using intracortical microstimulation (ICMS) to evoke peripheral movements (Hall and Lindholm, 1974; Donoghue and Wise, 1982; Sanderson et al., 1984; Neafsey et al., 1986; Miyashita et al., 1994; Brecht et al., 2004; Tandon et al., 2008). Studies using ICMS to map MI cortex generally find that the whisker region is located medially, whereas the forelimb and hindlimb representations are located, respectively, more laterally and caudally. When ICMS is combined with cytoarchitectonic analysis, the whisker region is linked to the medial agranular (AGm) area, whereas limb representations are in lateral agranular (AGl) cortex (Brecht et al., 2004).

Several investigators have sought to define the MI whisker representation by using neuronal tracing techniques to identify the motor cortical regions that receive projections from primary somatosensory (SI) cortex (Hoffer et al., 2003, 2005; Aronhoff et al., 2010; Mao et al., 2011). By placing tracers in the SI whisker region and then reconstructing the terminal labeling in agranular cortex, these studies demonstrate that SI barrel cortex projects to a strip of MI cortex located approximately 1–2 mm anterior and lateral to bregma.

No study has ever used ICMS and neuronal tracing in the same animal to determine if both techniques produce corresponding results. Although many sensory cortical regions project to MI cortex (Reep et al., 1990; Colechio and Alloway, 2009), the relative topography of their projections to MI has not been examined. Both the secondary somatosensory (SII) cortex and the posterior parietal cortex (PPC), for example, convey sensory information to MI, but how these projections relate to SI inputs or the motor maps produced by ICMS remains unknown.

In this study, we used multiple techniques to characterize the whisker representation in rat MI cortex. In some rats, one or two anterograde tracers were placed in the SI, SII, or PPC whisker regions to compare the topography of their projections to MI with respect to the cytoarchitectonic boundary that separates AGm from AGl. In some rats, the sensory innervation patterns were compared to the ICMS sites that evoked whisker movements. In other rats, two electrodes were placed in MI cortex, one at the border between AGm and AGl, and another more medially within AGm proper. Each electrode recorded isolated MI neuronal activity during controlled deflections of the peripheral whiskers, and then ICMS was administered at each electrode to compare their effectiveness at evoking whisker movements. The final set of animals received tracer injections in whisker-responsive parts of the superior colliculus (SC) to determine if corticotectal projections correspond to the motor-output region defined by ICMS.

The results show that projections from SI and SII terminate in a narrow cortical region located at the border between AGm and AGl. By comparison, the PPC projects to a narrow region in AGm that adjoins the area that receives projections from SI and SII. Consistent with this anatomical specificity, neurons in the transitional region between AGm and AGl are more responsive to whisker stimulation than neurons located more medially in AGm proper. By comparison, ICMS in the AGm is more effective in evoking whisker movements than at the AGm–AGl transition region. Finally, corticotectal projections that convey motor information from MI are preferentially clustered in AGm and AGl, but are sparsely represented in the transitional region between these areas.

## Materials and methods

Neuronal tracer injections and physiology experiments were conducted in male Sprague-Dawley rats (Charles River Co., Wilmington, MA) ranging in weight from 240 to 475 g. All procedures complied with NIH guidelines and were approved by our Institutional Animal Care and Use Committee.

### Animal surgery

Rats were anesthetized with an IM injection of ketamine HCl (40 mg/kg) and xylazine (12 mg/kg), and were subsequently administered atropine methyl nitrate (0.5 mg/kg), dexamethasone sodium phosphate (5 mg/kg), and enrofloxacin (2.5 mg/kg). Following an initial dose of ketamine and xylazine, rats were maintained in a lightly-anesthetized state by isoflurane (0.5–1.0%) for the remainder of the surgery. Anesthetic state was monitored by electrocorticography (ECoG) via a screw over the frontal cortex contralateral to the experimental hemisphere. Online Fourier analysis of the ECoG signal was used to identify the dominant frequency of cortical activity, and isoflurane levels were adjusted throughout the procedure to maintain the animal in a III-2 or III-3 stage of anesthesia (Friedberg et al., 1999).

Rats were intubated and secured in a stereotaxic frame (David Kopf Instruments, Tujunga, CA), and ventilated with oxygen. Heart rate, blood oxygen, and end-tidal carbon dioxide were monitored continuously during the procedure. Body temperature was maintained at 37.0°C, and ophthalmic ointment was applied to the eyes to prevent drying. A 2% solution of mepivacaine was injected into the scalp for local anesthesia, a midline incision exposed the cranial surface, and a ground screw was placed in the posterior cranium. Relative to bregma, craniotomies were made at coordinates that exposed SI (0–4 mm caudal and 3–7 mm lateral), SII (0–4 mm caudal and 6–9 mm lateral), PPC (1–5 mm caudal and 3–8 mm lateral), or SC (5–8 mm caudal and 1–3 mm lateral).

### Tracer injections

Anterograde tracers were placed in the whisker regions of SI, SII, or PPC. The tracers were either a 15% solution of biotinylated dextran amine (BDA, Invitrogen) or a 15% solution of Fluoro-Ruby (FR, Invitrogen). Locations of corticotectal projection neurons in MI were determined by injecting a combined solution of 2% Fluorogold (FG) and 15% BDA into whisker responsive sites in the SC.

To identify whisker-responsive sites in SI and SII, a glass pipette (~1 MΩ) filled with 3M saline was inserted into cortical layer IV (400–800 μm deep) at 25° from the sagittal plane. A recording wire was inserted into the saline and then connected to the headstage of an extracellular amplifier (Dagan 2200; Dagan Corp., Minneapolis, MN) to monitor neuronal activity (filtered 300–3000 Hz, 60 Hz notch) on a digital oscilloscope (Tektronix DPO4034; Tektronix Beaverton, OR) and an audio monitor. Receptive field mapping was performed with a wooden rod to identify SI barrel cortex as described in previous reports (Hall and Lindholm, 1974; Hoffer et al., 2003). To identify SII, the pipette was moved lateral to the A-row representation in SI. Similar to previous findings (Hoffer et al., 2003), we observed a mirror image representation of the SI whisker rows, and more ventral whisker representations were encountered as the electrode was marched further laterally.

To identify PPC, the pipette was moved caudal to the α, β, γ, and δ whisker arc in SI, where another mirror-image reversal of somatotopic organization was observed. Compared to SI, in which a principal whisker was identified at each recording site, multi-whisker receptive fields were observed in SII and PPC. Early reports on the PPC area in rats called it the parietal medial (PM) region to be consistent with prior descriptions of the somatosensory areas in the squirrel (Krubitzer et al., 1986; Koralek et al., 1990; Fabri and Burton, 1991), but subsequent studies emphasized the similarity of this region with the PPC region in primates (Reep and Corwin, 2009).

Another set of rats received tracer injections in whisker-responsive regions of the SC. A tracer-filled pipette was inserted orthogonal to the pial surface, and neuronal responses to mechanical whisker stimulation were recorded 4–5 mm deep. Additionally, ICMS was used to identify SC sites that evoke whisker movements as in other studies (McHaffie and Stein, 1982; Hemelt and Keller, 2008).

Deposits of BDA and FG/BDA were made using iontophoretic currents (2.5–5.0 μA, 10–20 min) administered on a 7 s on/off duty cycle through glass pipettes with tip diameters of 30–50 μm. Small volumes (120 nl) of FR were pressure injected through a glass pipette cemented onto the end of a Hamilton syringe. Following tracer injections, the rats were sutured and allowed to recover for 7–9 days before being sacrificed.

### Intracranial microstimulation

In MI cortex, saline-filled pipettes or tungsten electrodes were inserted orthogonal to the pial surface to a depth of ~1.5 mm, which corresponds to layer V. Trains of cathodal current pulses (0.7 ms duration) were administered at 250 Hz for 20, 40, or 80 ms at current levels ranging from 10–250 μA. In all rats, these current parameters produced brief muscle twitches that were easily visualized. When ICMS was tested at MI sites that evoked whisker movements, the stimulation always evoked rapid biphasic excursions characterized by retraction and then movement back to the resting position. When evoked responses were visualized, the identity of the responsive whiskers was recorded in the experimental protocol.

The EMG responses that accompany whisker movements evoked by ICMS were recorded in some rats. These recordings were obtained from two needle electrodes in the muscles of the whisker pad and a ground electrode in the musculature of the hindlimb. A Grass preamplifier (model P5; Grass Instrument Co., Quincy, MA) amplified and filtered the EMG signal (0.3–3000 Hz, 60 Hz notch), which was recorded by a DataWave Sciworks acquisition system (SciWorks, ver. 8.0; DataWave Technologies, Broomfield, CO) at a rate of 26 kHz. Raw EMG traces were used to measure latency from stimulus onset to the first motor response that exceeded baseline. The EMG records were low-pass filtered (250 Hz), rectified, and the mean response was calculated from 10 trials administered at each current level. The areal extent of the EMG envelope was used to quantify the magnitude of the muscle response.

### Extracellular neuronal recordings

Extracellular discharges recorded from MI were amplified (Dagan 2200; Dagan Corp., Minneapolis, MN), filtered, (300–3000 Hz), and converted into digital signals (DT2839, Data Translation, Marlboro, MA). Each neuronal recording channel was sampled at a rate of 26 kHz using the SciWorks data acquisition system. Waveforms were sorted using conventional parameters (spike height, width, valley time, and peak time), time-stamped at a resolution of 0.1 ms, and displayed as peristimulus time histograms (PSTHs).

### Whisker stimulation

Whisker deflections were administered by a Galvanometer from a Grass polygraph controlled by a digital waveform generator (ArbStudio; LeCroy, Chestnut Ridge, NY) activated by SciWorks. A small piece of window screen glued to the end of a pen on the Galvanometer was placed next to the rat's face so that vibrissa in rows A–E and arcs 1–5 protruded through the openings in the window screen. Each deflection consisted of a 50-ms back-and-forth pulse, first in the caudal direction (25 ms) and then back to the resting position (25 ms). In each trial, whisker stimulation consisted of three blocks of four deflections administered at frequencies of 2, 5, and 8 Hz. Because the first deflection in each block was preceded by a 1-s period, responses to the initial stimulus in each block were analyzed separately and classified as 1-Hz responses.

### Sacrifice

After each rat was deeply anesthetized with ketamine (80 mg/kg) and xlyazine (6 mg/kg), it was perfused transcardially with heparinized saline, 4% paraformaldehyde, and 4% paraformaldehyde in 10% sucrose. The brain was removed and stored in 4% paraformaldehyde with 30% sucrose in a refrigerator for 1–2 days.

### Histology

Prior to sectioning, the brainstem, cerebellum, and olfactory bulbs were removed, and the two hemispheres were separated. In some cases a cortical slab was removed from the hemisphere and flattened between two slides prior to being sectioned tangentially to enhance visualization of cytochrome oxidase (CO) in the SI barrel field. In other cases, the hemisphere was split into two parts along a coronal plane at bregma; this allowed the rostral forebrain to be sectioned coronally (to visualize MI cytoarchitecture) and the caudal part of the cortex to be sectioned tangentially (to visualize tracer injections relative to CO-labeled barrels in SI). In rats that received tracer injections in the SC, the entire brain was sectioned coronally.

All blocks of neural tissue were cut into 60-μm thick sections and placed in 0.1 M phosphate buffered saline (PBS). Tangential sections through the layer IV barrel field of SI were processed for CO (Wong-Riley, 1979; Land and Simons, 1985), and the remaining layers were processed for tracer labeling. For other tissue blocks, alternate sections were processed for tracer labeling and Nissl material.

To reveal BDA labeling, sections were processed using nickel and cobalt enhanced peroxidase immunohistochemistry as described previously (Smith et al., 2012). Tissue sections were rinsed in 0.3% H_2_O_2_ and then 0.3% Triton X-100 in 0.1 M PBS. Sections were subsequently incubated for 2 h in an avidin-biotin horseradish peroxidase solution (Vector Novocostra Laboratories, Burlingame, CA) in 0.3% Triton X-100 in 0.1 M PBS. Following incubation, sections were rinsed twice in 0.1 M PBS and then incubated in 0.06% diaminobenzidine (DAB), 0.0005% H_2_O_2_, 0.05% NiCl_2_, and 0.02% CoCl_2_ in 0.1 M tris buffer (pH = 7.2) for 10 min. The DAB reaction was halted by rinses in 0.1 M PBS. The processed sections were next mounted on gel-dipped glass and dried overnight. All sections processed for CO, Nissl material, or tracer labeling were dehydrated in ethanol, defatted in xylene, and then coverslipped.

### Anatomical analysis

An Olympus BH-2 microscope equipped for fluorescent microscopy was used to analyze tracer labeling. An Accustage plotting system (St. Paul, MN) was used to make digital reconstructions of tracer labeling in MI relative to anatomical landmarks. The BDA-labeled terminals were viewed in brightfield illumination, and fluorescent FR-labeled terminals were visualized using a TRITC filter (41002; Chroma Technologies). Axonal varicosities were plotted because these represent en passant synapses (Voight et al., 1993; Kincaid and Wilson, 1996; Meng et al., 2004).

Tracer overlap was analyzed with a module in the Accustage software. Each digital reconstruction was subdivided into a grid of square bins (25 and 50 μm^2^ were both tested), and each bin was color-coded blue if it contained BDA-labeling, red if it contained FR-labeling, or white if it contained both tracers. Tracer overlap was expressed as the proportion of tracer-filled bins that were colored white. Photographs of anatomical landmarks and tracer labeling were acquired using either an Epson V330 flatbed scanner or a Retiga EX CCD digital camera (Q-imaging, Surry, British Columbia, Canada).

## Results

A total of 18 rats were used in this study. The first 10 animals, listed in Table 1, received tracer injections in whisker-sensitive parts of SI, SII, and PPC to characterize the topography of their projections to MI. Another six rats were used in electrophysiology experiments in which MI neurons were recorded during whisker stimulation and then the recording sites were tested with ICMS. The final two rats received tracer deposits in whisker-related sites in the SC.

**Table 1:** **Summary of tracer injections**.

**Case**	**Tracer**	**Region**	**Tracer**	**Region**	**Plane of section**
FIN-1	BDA	SI			Tangential
FIN-2	BDA	SI			Coronal
FIN-3	BDA	SII			Coronal
FIN-4	FR	SI	BDA	SII	Tangential
FIN-5	BDA	SI			Coronal
FIN-6	BDA	PPC			Coronal
FIN-7	BDA	SI	FR	PPC	Tangential
FIN-8	FR	SI	BDA	SII	Coronal
FIN-9	FR	SI	BDA	PPC	Coronal
FIN-10	BDA	SI	FR	PPC	Coronal

### Topography of the SI projections to MI

**Figure 1:**
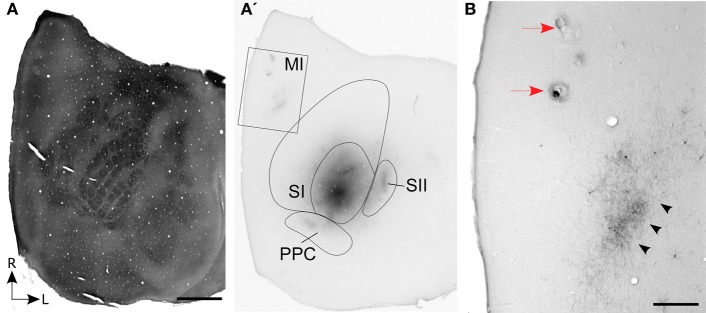
**Corticocortical projections from SI barrel cortex terminate at MI sites located lateral to the sites most effective for evoking whisker movements. (A)** Tangential section processed for cytochrome oxidase (CO) shows the spatial distribution of the layer IV barrels in SI cortex. **(A′)** An adjacent section processed for biotinylated dextran amine (BDA) shows the location of BDA deposits in SI barrel cortex. Contour lines indicate the primary (SI) and secondary (SII) somatosensory cortical areas as well as the posterior parietal cortex (PPC). Rectangle indicates the region depicted in panel **(B)**. **(B)** Location of two electrolytic lesions (red arrows) marking where intracranial microstimulation (ICMS) was most effective in evoking whisker twitches. Labeled projections (arrowheads) from SI terminate in a strip of MI cortex located caudal and lateral to sites that evoked the best whisker responses. Scale bars: 2 mm in **(A)**; 500 μm in **(B)**.

In three rats, the whisker-related projections from SI to MI were characterized by sectioning the entire cortex tangentially. In one case, which is illustrated in Figure 1, multiple BDA deposits were placed in the C-row of SI barrel cortex. The BDA deposits were confined entirely within the SI barrel field and, consistent with previous reports (Hoffer et al., 2003; Lee et al., 2011), revealed labeled projections to neighboring whisker regions in SII and PPC. In addition, labeling of the long-range projections from SI produced dense terminal labeling in a strip of MI cortex located approximately 2 mm from the midline.

On the day of sacrifice, ICMS was used to locate the MI sites most effective for evoking movements of the C-row whiskers. Microstimulation of MI sites located more than 2 mm lateral to the midline evoked twitches in the forelimb, shoulder, and neck muscles. Medial to these sites, low-threshold (~60 μA) currents evoked movements of the E and D-row whiskers. When the stimulating electrode was marched further medially, C-row whisker movements were evoked at the lowest currents (<50 μA) used in this experiment. Because these ICMS sites evoked movements from the same whiskers whose representations were filled with tracer deposits in SI barrel cortex, electrolytic lesions were made at these MI sites and the electrode penetrations were marked with ink. As indicated by Figure 1B, the labeled projections from SI barrel cortex terminated in a strip of MI cortex located caudal and lateral to the MI lesions that marked the locations for evoking movements of the C-row whiskers.

### MI cytoarchitecture

**Figure 2:**
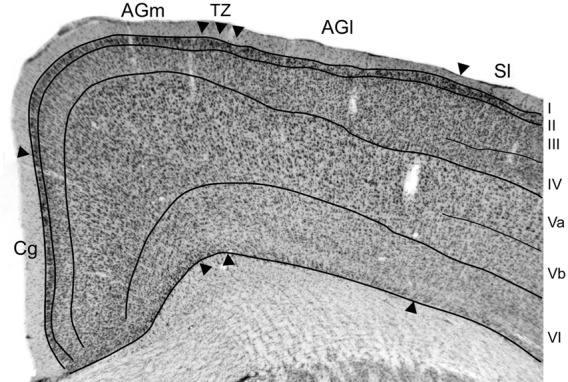
**Photomicrograph depicting the cytoarchitecture of MI cortex located 1.5 mm rostral to bregma.** The transitional zone (TZ) between the medial (AGm) and lateral (AGl) agranular areas is indicated by arrowheads. Additional arrowheads indicate the MI borders with the cingulate (Cg) and somatosensory (SI) cortical areas. Thin lines indicate laminar boundaries for layers I–VI.

The somatosensory cortical areas that received tracer deposits were always sectioned tangentially, but MI cortex was usually sectioned coronally so that we could examine its cytoarchitecture with respect to labeled projections from SI and its surrounding cortical fields. Figure 2 depicts the cytoarchitecture of MI cortex and the laminar changes that characterize the transition from AGm to AGl. Consistent with previous studies of rat motor cortex (Donoghue and Wise, 1982; Brecht et al., 2004), AGm has a relatively thick layer V that is dense with pyramidal neurons. More superficially, layer III is thin and has a noticeable pale appearance. Moving laterally toward the transitional zone between AGm and AGl, layer III gradually becomes much thicker as its neuronal density increases. By comparison, layer V becomes narrower as the bottom of layer III expands ventrally, and the neuronal density of layer V is much lower in AGl than in AGm. Layers III and V have a similar thickness in AGl, and the lateral edge of AGl is delineated by the sudden appearance of a dense granular layer IV, which signifies the medial edge of SI cortex.

### SI projections and MI topography

**Figure 3:**
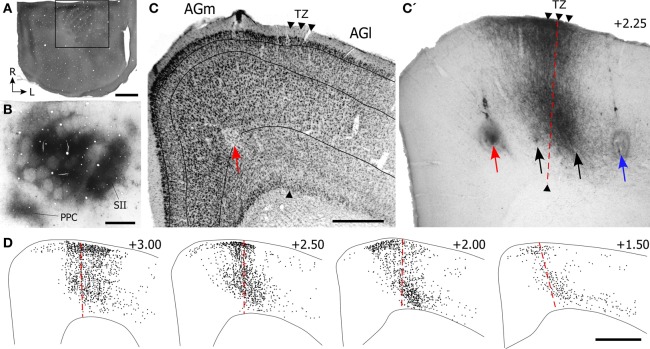
**Projections from SI barrel cortex terminate in the cortical area that marks the transition between AGm and AGl. (A)** CO-processed tangential section through the SI barrel field. The rostral half of the hemisphere is not apparent because it was sectioned coronally. Rectangle depicts the region in panel **(B)**. **(B)** Adjacent tangential section shows multiple BDA deposits in SI, as well as transport of BDA into SII and PPC. **(C,C′)** Adjacent sections processed for Nissl material and BDA-labeled projections from the SI tracer injections shown in panel **(B)**. The border between AGm and AGl is marked by arrowheads. Arrows indicate ICMS sites for evoking peripheral movements. Red arrow indicates the site where the lowest current (10 μA) evoked whisker twitches; black arrows indicate where higher currents (100–250 μA) evoked whisker movements; blue arrow indicates the ICMS site (50 μA) that evoked forelimb movements. **(D)** Plotted reconstructions illustrating labeled SI terminals with respect to the AGm–AGl border. Numbers indicate the distance from bregma in mm. Scale bar = 2 mm in **(A)**; 1 mm in **(B,D)**; 500 μm in **(C)**.

Experimental results depicting the topography of SI projections to MI are illustrated in Figure 3. After briefly mapping whisker-responsive sites in SI, multiple BDA deposits were placed so that the tracer infiltrated large portions of the SI barrel region (see Figure 3B). Local transport of the tracer revealed dense terminal labeling in SII cortex and in a smaller caudal area that represents PPC.

In MI cortex, a large bundle of labeled projections from SI coursed rostrally through cortical layer VI and abruptly turned toward the pial surface to form a vertical column of labeling (Figure 3C′). The labeled column was 400–500 μm in width and extended upwards from upper layer VI to layer I. Inspection of the adjacent Nissl-stained section indicated that this labeling was located in the MI region that represents the transition from AGm to AGl. As noted previously (Brecht et al., 2004), the border between these regions is often characterized by a gradual transition that can extend over several hundred microns.

Microstimulation was used to analyze the functional topography of MI with respect to the labeled projections from SI. After using saline-filled glass pipettes to evoke muscular responses at several sites within a single coronal plan, a tungsten electrode was reinserted at selected sites to verify each ICMS response and make a small electrolytic lesion. The locations of four microlesions that marked ICMS sites associated with whisker or limb movements are illustrated in Figure 3C.

The site at which the lowest stimulation current (10 μA) evoked whisker motion was in AGm, approximately 1.25 mm from the midline. Threshold currents for evoking whisker movements increased as the electrode moved to sites 1.75 mm (100 μA) and 2.15 mm (250 μA) lateral to the midline. The most lateral site, located 2.65 mm from the midline, was located in AGl and evoked dorsoflexion of the forepaw when a threshold current of 50 μA was administered. The vertical column of densely-labeled projections from SI, which occupied the AGm–AGl transitional zone, overlapped the two microlesion sites where whisker movements were evoked by moderately large current levels (100–250 μA). The most medial microlesion, which marked the site where whisker movements were evoked by the lowest stimulation currents (10 μA), was located in AGm proper.

Similar topographical responses were observed in other cases in which ICMS was systematically tested at different mediolateral locations in MI cortex. In all cases, low current levels near threshold evoked movements of either the whiskers or the forelimb in AGm and AGl, respectively. When suprathreshold currents were administered, however, movements of both whiskers and forelimb were occasionally observed, but only when the electrode stimulated the border between these functionally-defined regions.

Reconstructions of the terminal labeling patterns indicate that SI projections terminate in vertical columns that gradually move laterally as sections were analyzed from progressively more rostral parts of MI (Figure 3D). This medial-to-lateral shift is consistent with the diagonally-oriented strips of labeling observed in our tangential sections of MI cortex (Figure 1B), and it matches the systematic changes in AGm width observed by others (Donoghue and Wise, 1982; Tennant et al., 2011).

### SII projections and MI topography

**Figure 4:**
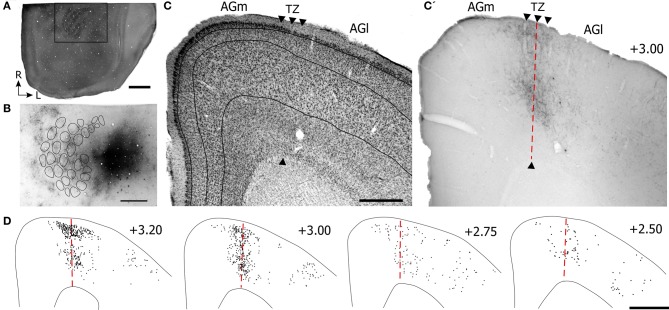
**Projections from SII cortex terminate in the transitional region between AGm and AGl. (A)** Tangential section depicting the CO-labeled barrel field in SI. Rectangle indicates the area depicted in panel **(B)**. **(B)** Multiple injections of BDA into the SII whisker region located lateral to SI. Locations of the CO-labeled barrels in SI are indicated by contours. Subsequent panels are illustrated as in Figure 3. Scale bar = 2 mm in **(A)**; 1 mm in **(B,D)**; 500 μm in **(C)**.

In three rats, anterograde tracing was used to determine the topography of SII projections with respect to the cytoarchitecture of MI (see Table 1). After whisker-induced responses were recorded at multiple sites in SI barrel cortex, the electrode was marched laterally to establish a reversal in the whisker map as successive sites in SII cortex were recorded. Once the C-row region in SII was located, BDA was injected so that it infiltrated much of SII without invading SI. As seen in Figures 4A,B, the BDA injection did not diffuse into the barrel field of SI, but many retrogradely-labeled neurons were present in both the septal and barrel columns as previously reported (Chakrabarti and Alloway, 2006).

Inspection of the labeled terminals in MI indicates that SII projects to the transitional zone between AGm and AGl (Figures 4C,C′), which is the same MI region that receives projections from SI (see Figure 3C′). However, despite the relatively large tracer injection in SII, the labeled projections to MI appeared weaker and were less extensive than the terminal labeling observed after tracer injections in SI. Compared to projections from SI, the SII projections innervated layers II, III, and the lower part of layer V of MI, but were weaker in upper layer V and did not innervate any part of layer VI. Furthermore, the SII projection terminals were more concentrated in the rostral half than in the caudal half of MI (Figure 4D).

**Figure 5:**
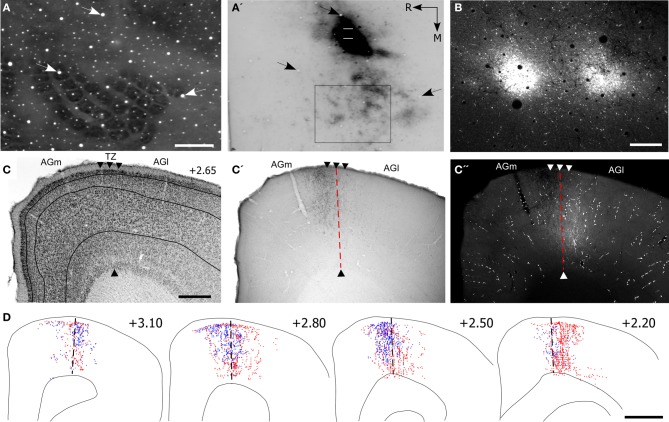
**Projections from the whisker regions in SI and SII terminate in the AGm–AGl transition zone. (A,A′)** Adjacent tangential sections depicting the SI barrel field **(A)** and the location of BDA deposits in SII **(A′)**. Arrows indicate common blood vessels. Rectangle around the SI barrel field indicates the region depicted in panel **(B)**. **(B)** Deposits of Fluoro-Ruby (FR) in SI barrel cortex. **(C)** Cytoarchitecture depicting the transition zone (arrowheads). **(C′,C′′)** Adjacent sections depicting the terminal projections from SII and SI with respect to the transition zone (dashed line). **(D)** Reconstructions of SI (red) and SII (blue) labeled terminals throughout the rostrocaudal extent of MI. Scale bar = 500 μm in **(A,C)**; 250 μm in **(B)**; 1 mm in **(D)**.

To confirm that both SI and SII innervate overlapping regions, paired injections of different anterograde tracers were placed in SI and SII. As shown by Figure 5, injections of FR into the C and D rows of SI were paired with a BDA injection in the C-row representation of SII. In both cortical areas, tracer injections were guided by mapping neuronal responses to whisker stimulation. Tracer placements in SI and SII were visualized in tangential sections processed for CO, and microscopic examination confirmed that corresponding whisker representations were injected in both areas as indicated by the presence of BDA-labeled neurons at the FR injection site (Figures 5A′,B).

The labeled projections from SI and SII terminated in an overlapping part of MI located in the transitional region between AGm and AGl (Figures 5C′,C′′). While the labeled projections from SI infiltrated all layers of MI, those from SII were noticeably absent from layer VI. Nonetheless, reconstructions of terminal labeling in MI revealed substantial tracer overlap in the transitional region between AGm and AGl. This overlap extended throughout MI cortex but was densest in its rostral half (Figure 5D).

### PPC projections to motor cortex

To characterize more completely the sensory-related cortical regions that innervate MI cortex, we injected PPC with anterograde tracers and analyzed the labeled projections with respect to the AGm–AGl border. These experiments were prompted by previous studies showing that PPC projects to AGm but not to AGl (Reep et al., 1990; Colechio and Alloway, 2009).

**Figure 6:**
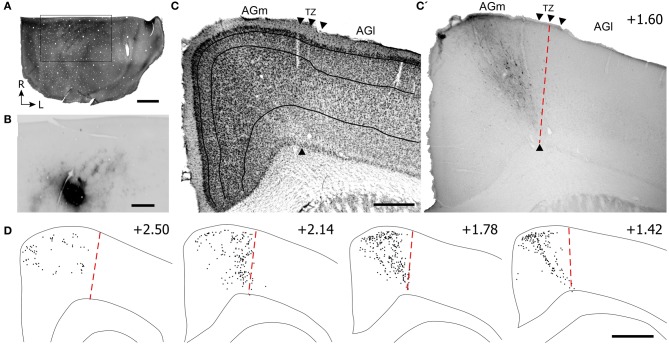
**Projections from posterior parietal cortex (PPC) terminate primarily in the caudal part of AGm. (A,B)** Adjacent tangential sections showing CO-labeled barrels in SI and the BDA tracer deposit in PPC, which is caudal to SI. Rectangle indicates the magnified image of the PPC injection site and BDA labeling in the SI septa. **(C,C′)** Adjacent sections depicting the labeled projections from PPC with respect to the transition zone. Arrowheads indicate cytoarchitectonic boundaries. **(D)** Plotted reconstructions indicate PPC projections terminate most densely in caudal parts of MI. Scale bar = 2 mm in **(A)**; 1 mm in **(B)**; 500 μm in **(C)**; 1 mm in **(D)**.

To locate the whisker region in PPC, neuronal responses to whisker stimulation were initially tested in the caudal part of SI barrel cortex. Sites located caudal to SI were then tested, and neurons in this area had multi-whisker receptive fields. As indicated by Figure 6, tracer deposits in PPC were caudal to the CO-labeled barrels in SI. Although the tracer did not diffuse into SI, retrogradely-labeled neurons and other processes appeared in the SI septal regions as described earlier (Lee et al., 2011).

Virtually all labeled projections from PPC were located in AGm proper (see Figures 6C,C′). In each coronal section that was inspected, the PPC projections terminated in the region medial to the AGm–AGl transition zone (Figure 6D). The labeled terminals were intermingled with labeled neuronal soma, which indicates that PPC and AGm are reciprocally connected. In contrast to SII, the PPC projections were concentrated in the caudal half of MI.

**Figure 7:**
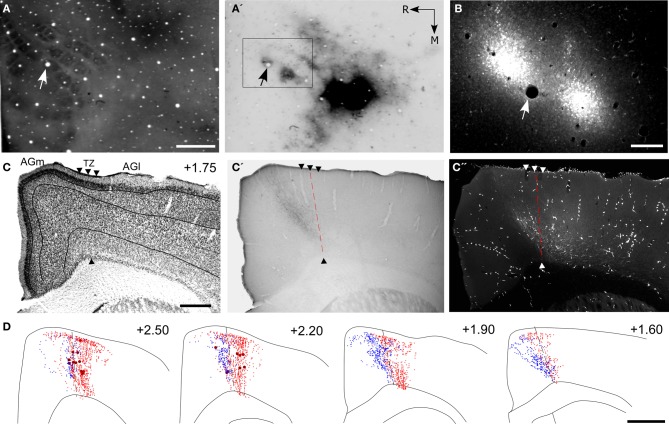
**Projections from PPC and SI terminate in adjoining MI regions. (A,A′,B)** Tangential sections showing CO-labeled barrels and tracer injections in PPC and SI as in Figure 5. Arrows indicate common blood vessels. **(C,C′,C′′)** Adjacent coronal sections illustrate labeled projections from PPC **(C′)** and SI **(C′′)** with respect to the transition zone. **(D)** Reconstructions of the labeled terminals in MI originating from PPC (blue) and SI barrel cortex (red). Scale bars = 500 μm in **(A,C)**; 250 μm in **(B)**; 1 mm in **(D)**.

To verify that SI and PPC innervate adjacent parts of MI, we placed pairs of different anterograde tracers in SI and PPC of three rats (see Table 1). Figure 7 illustrates a case in which FR and BDA were placed in whisker-sensitive sites in SI and PPC, respectively. Histological inspection revealed that many SI neurons retrogradely-labeled by the PPC tracer injections were at sites that overlapped the tracer injections in SI (Figures 7A′,B), thereby indicating that both tracers were injected into corresponding whisker regions.

These dual tracer experiments indicate that SI and PPC innervate adjoining, partially overlapping regions in MI. The projections from PPC innervate regions located medial to most of the MI sites that receive SI inputs. While SI projections terminate densely in the AGm–AGl transition region (Figure 7C′′), the labeled projections from PPC terminate almost exclusively in an adjoining part of AGm proper (Figure 7C′). Reconstructions of labeled terminals throughout the rostrocaudal extent of MI depict some overlap among the projections from PPC and SI (Figure 7D), but most of the axons labeled by different tracers terminated in separate regions. Consistent with this distinction in projection targets, the PPC projections are densest in caudal MI, whereas those from SI are densest in rostral MI.

### Overlap analysis

To assess the amount of convergence among SI, SII, and PPC projections to MI, we quantitatively analyzed the labeled overlap produced in the five rats that received paired tracer injections (see Table 1). For this purpose, plotted reconstructions of the MI terminal labeling patterns were subdivided into square bins that were color-coded according to the type of terminal labeling present in each bin. Then, in each of the five rats, the proportion of all bins throughout MI that contained tracer overlap was measured so that overlap resulting from SI and SII tracer injections could be compared with the overlap produced by injections in SI and PPC.

**Figure 8:**
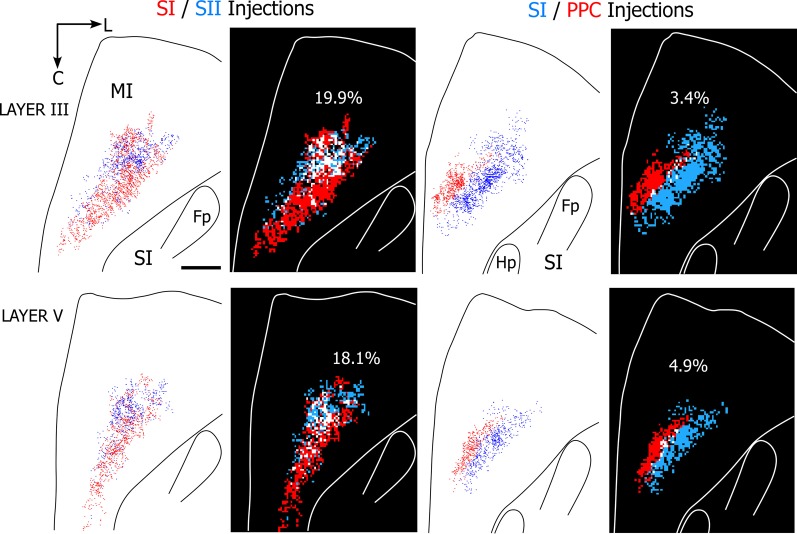
**Projections from SI and SII overlap in MI, but projections from SI and PPC terminate in adjoining MI areas. Left panels** depict results from a rat in which SI and SII were injected with FR (red) and BDA (blue), respectively. **Right panels** depict results from a second rat in which SI and PPC were injected with BDA and FR, respectively. In both cases, the panels depict labeling patterns in tangential sections obtained from layers III or V. For each section, the plotted reconstructions of the labeled terminals were subdivided into square bins (50 μm^2^) color-coded by whether the bin contains terminal projections from one injection site (blue or red) or overlapping projections from both injections (white). Amount of overlap in each section is indicated by percentages. Scale bar = 1 mm. Abbreviations: HP, hindpaw; FP, forepaw.

The results indicate that projections to SI and SII overlap substantially more than the projections to SI and PPC regardless of whether 25 or 50 μm^2^ bins were used in the analysis. Figure 8 illustrates the amount of overlap in tangential sections obtained from two rats in which different tracers were injected either into SI and SII or into SI and PPC. As indicated by these individual sections, which depict 50 μm^2^ bins, projections from both SI and SII were present in nearly 20% of the labeled area, whereas overlapping projections from SI and PPC occupied less than 5% of the labeled area. When tallied across all sections in each rat, MI tracer overlap in the SI and SII cases (*n* = 2) varied from 16.4% to 26.9%, but ranged from only 2.5% to 6.9% in the rats that received injections in SI and PPC (*n* = 3). Although the number of dual tracer cases is too small for a rigorous statistical comparison, the large differences in overlap between these groups suggest that PPC projects to an MI area that is separate from the area innervated by SI and SII.

### Topography of sensory responses in MI

The overlap of SI and SII projections to the AGm–AGl transitional zone suggests that this region is specialized for processing sensory inputs. To confirm that SI and SII conveys whisker-related information to this region, we recorded neuronal responses at the AGm–AGl border during controlled whisker deflections. Furthermore, to determine if this region is functionally distinct from AGm proper, we recorded neurons in both areas simultaneously so that we could compare their whisker-induced responses. For this purpose, two electrodes were independently inserted into layer V of both regions because previous work showed that MI responses to whisker stimulation are maximal at sites located 1–1.5 mm below the pial surface (Chakrabarti et al., 2008). In addition, after recording neuronal responses to whisker deflections at each electrode, we administered ICMS at each recording site to determine if both regions are equally effective in evoking whisker movements.

**Figure 9:**
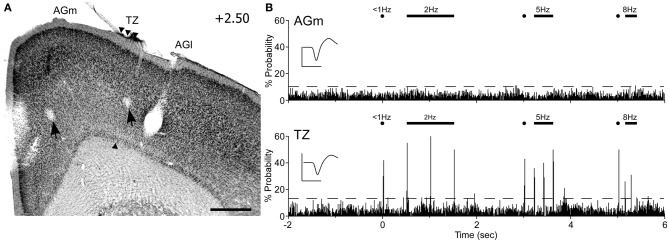
**Neuronal responses in MI during computer-controlled whisker deflections. (A)** Coronal section through MI showing two recording sites (arrows) with respect to the transition zone. Scale bar = 500 μm. **(B)** Peristimulus time histograms (PSTHs) show the responses recorded in AGm (top) and the transition zone (bottom). Controlled stimulation consisted of 50-ms deflections applied simultaneously to 15 whiskers at multiple frequencies over 200 trials. Dashed lines represent 99% confidence interval. Waveform scales: 200 μV and 1 ms. PSTH bins: 10 ms.

Peripheral whisker stimulation evoked strong neuronal responses in the AGm–AGl transition zone, but did not activate neurons in AGm proper. As illustrated by Figure 9, spontaneous discharge rates were similar for the neurons recorded in each area. Yet, whereas the neuron in the transition zone responded to each tested frequency (2, 5, and 8 Hz), the neuron in AGm did not respond to any whisker deflections. Subsequent administration of ICMS at each recording site evoked whisker movements that were easily visualized.

In all rats (*n* = 6), histology confirmed that whisker-responsive neurons were recorded in the AGm–AGl transition zone, which coincides with the afferent projections from SI and SII. A total of nine pairs of MI neurons were recorded, and in each case the AGm neuron failed to show an increase in discharge rate when the whiskers were deflected. One AGm neuron, however, displayed spontaneous activity that was inhibited when the whiskers were stimulated. All neurons in the AGm–AGl transition zone were activated by whisker stimulation. While some responded at each frequency of whisker stimulation (4/9), the rest responded to stimulation up to 5 Hz (5/9). These results indicate that the transitional region between AGm and AGl represents a functionally distinct part of MI that is specialized for processing sensory inputs.

### Comparison of sensory and motor responses

Some of the electrophysiology experiments (*n* = 3) were conducted in rats in which we compared the sensory-input and motor-output functions of AGM and the AGm–AGl transitional zone. As in the other MI recording experiments, one electrode was placed in AGm and the other was placed at the AGm–AGl border to record neuronal responses in both regions at one or two depths during controlled whisker deflections. Subsequently, for ICMS, the electrodes were advanced to deep layer V (1.5 mm) where corticobulbar neurons originate. Microstimulation was alternately administered to each electrode on every trial, and EMG responses were recorded by needle electrodes inserted into the whisker pad (see “Materials and Methods”).

**Figure 10:**
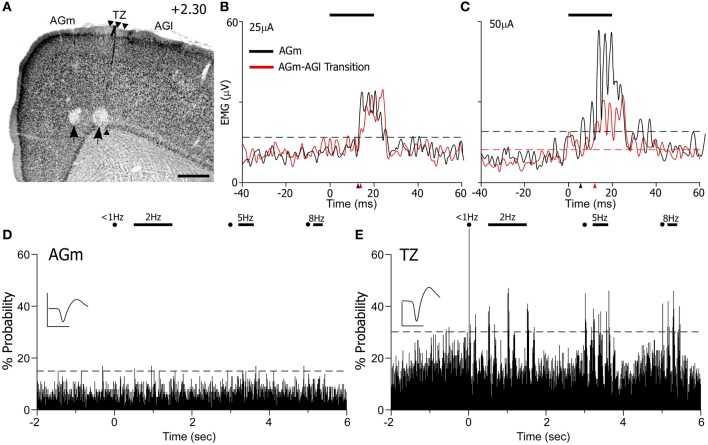
**ICMS-evoked whisker movements are strongest at AGm, but sensory-evoked responses are present only at the transition zone. (A)** Nissl-stained section showing recording sites marked by lesions (black arrows). Scale bar = 500 μm. **(B,C)** EMG responses to ICMS (bar) administered at two current levels in AGm or the transition zone. Each graph represents the mean of 10 trials. Arrowheads indicate onset of EMG response; dashed lines represent maximum pre-stimulus activity. **(D,E)** PSTHs illustrate sensory-evoked responses as in Figure 9. Waveform scales: 200 μV and 1 ms. PSTH bins: 10 ms.

Administration of ICMS evoked detectable differences in EMG responses that varied with the location of the MI stimulating electrode. For the case shown in Figure 10, ICMS with currents of 10 μA evoked whisker movements only in AGm on 20% of the trials. When higher current (25 μA) levels were employed, both AGm and the transition zone were effective in evoking EMG responses, but EMG responses evoked from AGm had shorter latencies than the responses evoked from the transition zone (see Figure 10B). The latencies measured from the raw EMG responses (10 trials) was 15.2 ms for AGm and 17.2 ms for the transition zone, consistent with latencies from other studies performing ICMS in MI (Berg and Kleinfeld, 2003). Although the peak amplitudes of the EMG responses evoked by both sites were similar at near threshold current levels, more whiskers responded to ICMS at AGm (whiskers in rows B–E, arcs 1–4) than at the AGm–AGl transition zone (whiskers in rows C–D, arcs 1–2).

At higher current levels (50 μA), ICMS at both cortical regions produced whisker retractions, but the EMG responses evoked from AGm had larger amplitudes (see Figure 10C). Consistent with this, the latency of the EMG responses evoked from the AGm electrode became even shorter when compared to the latency of the responses evoked from the AGm–AGl border (14.6 vs. 16.7 ms). In fact, in the three cases analyzed, EMG responses evoked by ICMS at AGm had shorter latencies (16.3 ± 0.3 ms) than the EMG responses evoked from the AGm–AGl border (17.3 ± 0.4 ms). Compared to the AGm–AGl transition zone, these findings suggest that AGm proper has stronger functional connections with the periphery whisker pad.

In experiments in which EMG responses were recorded during ICMS, the MI responses to controlled whisker stimulation had already been recorded from neurons encountered before the electrodes reached deep layer V. As before, whisker-induced sensory responses were recorded from neurons in the AGm–AGl transition zone but not from neurons in AGm proper (see Figure 10D). Hence, whisker-evoked responses in the AGm–AGl transition zone are consistent with the specificity of the projections from SI and SII. Coupled with the differential EMG responses evoked by ICMS at these two MI regions, these data indicate that rat MI contains distinct sensory-input and motor-output subregions.

### Organization of corticotectal projections from MI

In an effort aimed at characterizing a route for conveying MI signals to the facial nucleus, we placed tracers into the whisker representation of the SC. This brain region was chosen because SC is one of the main targets of MI corticofugal projections and the SC projects directly to the facial nucleus (Miyashita et al., 1994; Alloway et al., 2010). In two rats, we injected a combined solution of anterograde (BDA) and retrograde (FG) tracers into SC sites that responded to whisker deflections and were effective at evoking whisker movements when tested with ICMS.

**Figure 11:**
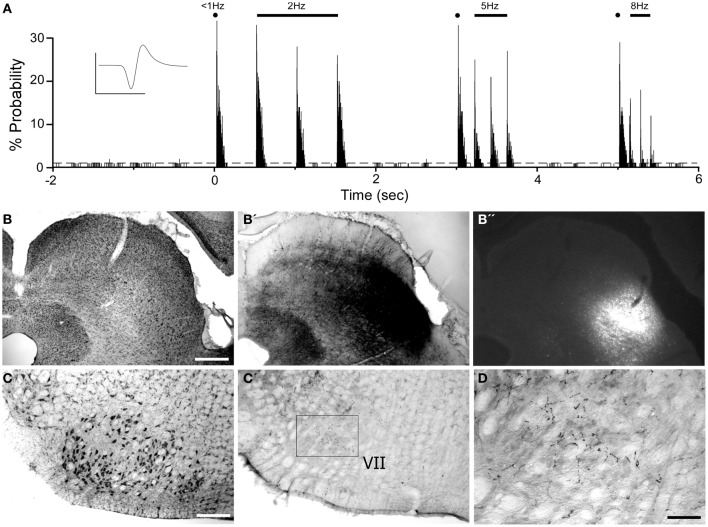
**Whisker-responsive region in the superior colliculus (SC) projects to the contralateral facial nucleus. (A)** PSTH illustrating the whisker-induced responses of a SC neuron recorded by a pipette containing FG and BDA. **(B)** Nissl-stained coronal section showing the laminar location of BDA **(B′)** and FG **(B′′)** injections at the neuronal recording site. **(C)** Nissl-stained section showing the facial nucleus contralateral to the SC injection site **(C′,D)** BDA-labeled terminals in the facial nucleus. Waveform scales: 200 μV and 1 ms. PSTH bins: 2 ms. Scale bars = 500 μm in **(B)** and **(C)**; 250 μm in **(D)**.

Consistent with previous work (Hemelt and Keller, 2006), neurons in the ventrolateral SC respond to repetitive deflections of the contralateral whiskers as shown in Figure 11. A combined injection of BDA and FG into this site produced limited diffusion of both tracers, but revealed many local projections within the SC and to the periaqueductal gray region. In addition to labeled projections to the reticular formation and spinal trigeminal nucleus (data not shown), BDA-labeled terminals also appeared in the contralateral facial nucleus (see Figures 11C,C′,D). This result confirms that the tracers were placed in a region that could transmit MI information to the facial nucleus.

**Figure 12:**
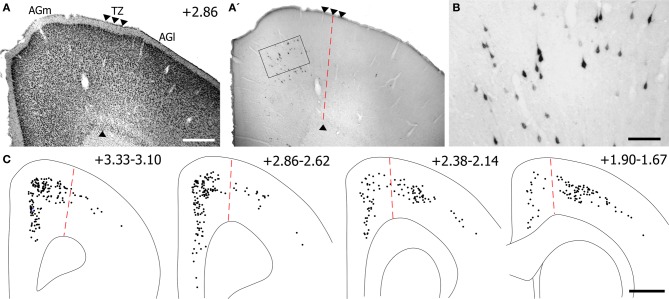
**Most corticotectal projections from MI originate from AGm. (A)** Nissl-stained section of MI showing boundary between AGm and AGl. **(A′,B)** Photomicrographs illustrating MI neurons retrogradely-labeled by the BDA tracer injection shown in Figure 11. Corticotectal neurons are densest in AGm. Rectangle depicts region shown in panel **(B)**. **(C)** Digital reconstruction of labeled neurons throughout the rostrocaudal extent of MI. Two plotted reconstructions are superimposed in each depiction. Scale bars = 500 μm in **(A)**; 100 μm in **(B)**; 1 mm in **(C)**.

Corticotectal neurons labeled by tracer injections in SC were present throughout MI cortex. As seen in Figure 12, populations of labeled neurons were easily visualized in tissue sections processed for BDA. Although BDA is normally used as an anterograde tracer, injecting it in solution with FG greatly increases its retrograde transport (Smith et al., 2012). The topographic distributions of BDA-labeled and FG-labeled neurons were identical, but we chose to plot and photograph only BDA-labeled neurons because these neurons were easier to visualize, especially at low magnifications.

The majority of corticotectal neurons labeled by tracer injections in whisker-sensitive parts of the SC are located in AGm proper. As illustrated by our plotted reconstructions, the densest clusters of labeled neurons appeared in AGm proper. Within some coronal sections, these large clusters of labeled neurons were easily visualized in AGm at low magnifications (see Figure 12A′). Secondary clusters of labeled neurons also appeared in AGl at specific rostrocaudal coordinates, which is consistent with previous work showing that the forelimb representation in AGl projects to scattered targets in the SC (Alloway et al., 2010). Compared to the main parts of AGm and AGl, the transition zone between these regions contained few labeled neurons. In fact, in many coronal sections the AGm–AGl transition zone was devoid of labeled neurons even though distinct clusters of corticotectal neurons appeared in AGm or AGl.

## Discussion

This study characterized the functional organization of the MI whisker representation by using a combination of techniques that included anterograde tracing from multiple cortical areas, paired neuronal recordings in MI during whisker stimulation, and recordings of EMG signals in the whisker pad during ICMS at different MI sites. The tracing results indicate that projections from SI and SII terminate in the transitional zone between AGm and AGl. Neurons in this region are highly responsive to whisker stimulation, but neurons in AGm proper do not respond to passive whisker deflections. By contrast, AGm is more effective for evoking whisker responses. These findings suggest the existence of functionally-distinct subregions within the whisker representation in MI cortex: a sensory-input region in the AGm–AGl transition zone and a motor-output region in AGm proper.

### Sensory processing in MI

Our findings suggest that MI cortex contains one or more subregions that are specialized for processing sensory inputs. The existence of a sensory processing region in MI was previously suggested by tracing studies showing that whisker-related areas in SI and SII project to a relatively compact part of MI (Hoffer et al., 2003, 2005; Colechio and Alloway, 2009; Aronhoff et al., 2010). Consistent with previous results (Chakrabarti et al., 2008), we confirmed that passive whisker deflections evoke neuronal responses at MI sites that receives sensory inputs from SI and SII.

Our results also suggest the possibility that MI cortex might contain more than one sensory processing region. In addition to the border region between AGm and AGl, the adjoining region in AGm receives inputs from PPC that display minimal overlap with the projections from SI. Tracing studies indicate that PPC receives somatosensory, auditory, and visual cortical inputs (Reep et al., 1994). Consistent with these multimodal sensory inputs, lesions that damage PPC or its connections with frontal cortex produce attention deficits characterized by hemispatial sensory neglect (Burcham et al., 1997; Reep and Corwin, 2009). Compared to projections from SI and SII, the anatomical and functional specificity of the PPC projections suggest that its target region in AGm might represent an additional MI area that is specialized for processing multimodal spatial information.

Axonal projections from SI to MI originate from the septal circuits in SI barrel cortex (Alloway et al., 2004; Chakrabarti and Alloway, 2006; Chakrabarti et al., 2008). Although the functions of the septa remain controversial, substantial evidence suggest that septal circuits encode information about the kinematics of active whisker movements (Alloway, 2008). In this context it is noteworthy that PPC derives its SI inputs from the septal circuits (Lee et al., 2011). This prompts the view that the AGm–AGl transition zone is responsible for processing relatively pure kinematic information received from SI, whereas the adjoining subregion in AGm proper processes whisking information that is integrated with visual and auditory inputs in the PPC. Presumably, both of these sensory-related MI regions provide information to AGm proper that helps refine the execution of the cortical motor programs that regulate whisking behavior.

### MI control of whisking behavior

The role of motor cortex in whisking behavior is a contentious topic. Some investigators who used ICMS in awake animals concluded that MI exerts direct control over specific aspects of whisking behavior (Berg and Kleinfeld, 2003; Haiss and Schwarz, 2005; Cramer and Keller, 2006). Consistent with this view, neuronal recordings during whisking behavior indicate that MI neurons “report the absolute angle of vibrissa position” even after the trigeminal nerve has been transected (Hill et al., 2011). By contrast, others have suggested that AGm “plays a role in orienting that is independent from any role in control of whisking” (Erlich et al., 2011). These conflicting views emphasize the need for using multiple approaches to elucidate the functional organization of the MI whisker region.

Our data indicate that ICMS in AGm proper is more effective for evoking whisker movements than ICMS in the AGm–AGl transition zone. Compared to the transition zone, we observed that microstimulation in AGm evokes movements from a larger number of whiskers when the same current levels were administered to both regions. These visual observations were usually confirmed when we compared the amplitudes of EMG responses recorded at the mystacial whisker pad. In addition, EMG responses evoked from AGm appeared earlier than the responses evoked from the AGm–AGl transition zone. Based on these differences in the EMG response latencies, we conclude that the outputs from AGm proper have stronger and more direct connections with the facial nucleus than the outputs from the AGm–AGl transition region.

Short-latency EMG responses evoked from AGm are consistent with tracing studies showing that AGm projects to many brainstem regions, including the SC, that project to the facial nucleus (Hattox et al., 2002; Grinevich et al., 2005; Alloway et al., 2010). This was corroborated by the present study, which used anterograde and retrograde tracing to demonstrate that whisker-sensitive regions in SC have input-output connections that could transmit MI information directly to the facial nucleus. Although projections originating exclusively from the AGm–AGl transition zone were not characterized, we found that most MI inputs to whisker-responsive sites in the SC originate from the AGm. Although the SC also receives MI inputs from AGl, very few corticotectal neurons were located in the AGm–AGl transition region. Therefore, the AGm–AGl transition region appears to be weakly connected with SC, and this could explain why the border region between AGm and AGl is less effective in evoking EMG responses.

An alternative explanation concerns the intrinsic connections within MI cortex. When tracers are placed in the center of the whisker (AGm) or forelimb (AGl) regions, the labeled connections remain within the injected region (Weiss and Keller, 1994). When tracers are placed at the border between these regions, however, labeled axons project throughout both the whisker and forelimb areas. Hence, when ICMS is applied to the border region between AGm and AGl, it may activate local projections to AGm that, in turn, activate the long-range outputs from AGm. Although the functional role of these local horizontal connections has not been established, they could provide a route by which the sensory processing subregion modulates AGm activity during exploratory whisking behavior.

### Functional subregions

Previous reports indicate that cortical control of whisking is parcellated into distinct subregions, but this view is mainly concerned with rostrocaudal distinctions in the kinematics of whisker movements evoked by ICMS (Haiss and Schwarz, 2005). To our knowledge, no study has demonstrated functionally-distinct subregions in the mediolateral dimension of MI. Even though previous work indicates that SI projections to MI do not terminate at coordinates that are optimal for evoking whisker movements (Brecht et al., 2004), the spatial relationship between sensory inputs and the effects of ICMS were never examined in the same animal. The present study indicates that SI and SII projections to MI terminate specifically in the AGm–AGl transition region, and this region is less effective than AGm for evoking whisker movements.

Our results suggest that MI whisker cortex contains a sensory-input region that is spatially segregated from the motor-output region. Conceivably, the whiskers and other body part representations in MI are comprised of distinct sensory-input and motor-output regions so that specific motor behaviors are executed by separate parts of MI cortex. For example, PPC inputs may terminate in whisker and neck regions of MI, as defined by ICMS, to facilitate the coordination of the motor elements that comprise orienting behavior. Likewise, SI projections to rostral MI may convey information about the whiskers and the nose representation to help coordinate sniffing and whisking behavior (Welker, 1964; Hoffer et al., 2003). Testing these hypotheses, however, requires the combination of tracing data and physiological recordings in awake behaving animals.

### Conflict of interest statement

The authors declare that the research was conducted in the absence of any commercial or financial relationships that could be construed as a potential conflict of interest.
